# The effect of preoperative aminophylline on the recovery profile after major pelvic-abdominal surgeries: a randomized controlled double-blinded study

**DOI:** 10.1186/s12871-021-01340-7

**Published:** 2021-04-19

**Authors:** Samaa A. Kasim, Mahmoud Hussein Bahr, Mohamed Abdelkader, Doaa Abu Elkassim Rashwan

**Affiliations:** Faculty of Medicine, Beni -Suef University, Beni - Suef, Egypt

**Keywords:** Aminophylline, Adenosine, BIS

## Abstract

**Background:**

This study compared the effects of premedication with different doses of aminophylline on the recovery profile after general anaesthesia.

**Methods:**

Forty-five patients scheduled for pelvic-abdominal surgeries were divided into 3 groups: Group C: the patients received 100 ml of **IV** normal saline, Group A1: the patients received 2 mg/kg IV aminophylline, and Group A2: the patients received 4 mg/kg IV aminophylline 30 min before induction of general anaesthesia. The following data were recorded: demographic data, ASA physical status, duration of anaesthesia and surgery, heart rate, mean arterial blood pressure, propofol dose, fentanyl dose, times to reach BIS (48 ± 2) after induction of anaesthesia and to reach a value of 80 after discontinuation of sevoflurane anaesthesia, time to recovery of consciousness and to tracheal extubation and to discharge from the post-anaesthesia care unit, and side effects of aminophylline.

**Results:**

The time to reach a BIS of 48 ± 2 was significantly lower for the control group than group A2 (70.67 ± 22.50 and 106.67 ± 34.77 s for groups C and A2, respectively, *p* -value =0.01). The time to reach a BIS of 80 was significantly longer for the control group than group A1 andA2 (5.6 ± 1.40,3.5 ± 1.93and 2.53 ± 1.72 min for groups C,A1 and A2, respectively, p -value < 0.01). The time to ROC was significantly longer for the control group than groups A1 and A2 (8.93 ± 0.92, 5.6 ± 2.47 and 4.53 ± 3.33 min for groups C, A1 and A2, respectively; *p* -value < 0.01). The extubation time was significantly longer for the control group than groups A1 and A2 (12.4 ± 1.08, 7.87 ± 3.27 and 6.6 ± 2.47 min for groups C, A1 and A2, respectively; *p* -value < 0.01).

**Conclusion:**

Premedication with aminophylline enhanced the recovery profile after pelvic-abdominal surgeries under general anaesthesia without cardiovascular complications.

**Clinical trial registration:**

Name of the registry: Register@ClinicalTrials.gov

Trial registration number: ClinicalTrials.gov Identifier: NCT04151381.

Date of registration, November 5, 2019, ‘Retrospectively registered’.

## Background

Aminophylline acts through either phosphodiesterase inhibition or by blockade of adenosine receptors [1]. Aminophylline antagonizes the effects of barbiturates [2], sevoflurane [3], and morphine [4]. However, these studies were uncontrolled clinical trials, which make it difficult to confirm the effects of aminophylline on the actions of hypnotics and anaesthetic agents. It has been reported that aminophylline delays loss of consciousness (LOC) and speeds recovery of consciousness (ROC) with propofol anaesthesia, but these results were found in volunteers in the study of Turan et al. [5]; the researchers administered saline or aminophylline at 6 mg/kg (IV) then 1.5 mg/kg/h (as a continuous infusion) followed by 200 mg propofol. In this study, the time to LOC was longer and the time to ROC was shorter in the aminophylline group then the saline group. In case reports presented by Sakurai et al. [6], where 5 mg/kg aminophylline reversed propofol-induced sedation in the postoperative period, there was controversy about the optimum dose of aminophylline since toxicity was reported when it was given at high doses or as a continuous intravenous infusion [7]. Some studies suggested that low doses of aminophylline (1–2 mg/kg) reversed the sedation induced by benzodiazepines, barbiturates or opioids [8]. Other reports recommended the use of higher doses (4–6 mg/kg) to effectively awaken anaesthetized patients because the lower doses produced an incomplete reversal of sedation or hypnosis [1, 6, 9–11]. Moreover, the available published results are from uncontrolled clinical studies, which makes it difficult to confirm the effects of aminophylline on hypnosis and recovery profiles, and Sakurai et al. [6] reported that further studies are needed to determine the appropriate doses for antagonizing sedation and to test the best method of aminophylline administration. Thus, based on these findings, and the conclusions of Turan et al. [5] that their results may differ with different patient samples or other clinical conditions and to the best of our knowledge, aminophylline has not been given as a pretreatment to test its effect on the level of hypnosis and recovery profile after general anaesthesia. Furthermore, it was recommended to evaluate the effective dose of aminophylline that affects the extubation and recovery times [5]. Since there is controversy about the effective dose of aminophylline, two different doses were selected [8, 10], 2 or 4 mg/kg [9]. Therefore, this study hypothesized that pretreatment with different doses of aminophylline may decrease the time to ROCs, extubation time and time to discharge from the postanesthesia care unit (PACU), and its effect on the level of hypnosis (using BIS) was also observed. To test this hypothesis, this controlled study examined the effects of 2 or 4 mg/kg aminophylline [10, 11] when given as a pretreatment in patients undergoing pelvic-abdominal surgeries under general anaesthesia compared to controls based on the time of ROC as the primary outcome of this study. The effects on time to reach a BIS value of 80 after sevoflurane discontinuation [12], the propofol dose (mg) until BIS reached 48 ± 2 s, time to tracheal extubation (in minutes) and time to discharge from the PACU (in minutes) were also assessed as secondary outcomes, and the side effects of aminophylline administration were observed and recorded.

## Methods

This randomized controlled double-blinded study was conducted at Beni-Suef University Hospital from 20 November 2019 to 25 February 2020 in compliance with the Helsinki Declaration after approval of the Research Ethical Committee of Beni-Suef University Hospitals (FMBSUREC/01102019/Rashwan) and obtaining written informed consent from the patients. The study was registered at ClinicalTrials.gov with identification number NCT04151381 and date November 5, 2019, retrospectively, and adhered to CONSORT guidelines. The study included 45 patients of both sexes in the age group of 20–60 years and American Society Of Anesthesiology physical status I-II who were scheduled for pelvic-abdominal surgeries under general anaesthesia**.** Patients were excluded if their body mass index was more than 30 kg/m2 or if they had sensitivity to aminophylline or history of seizure, renal or hepatic impairment or coffee consumption (more than 2 cups/day). Patients with opioid addiction or patients treated with B agonists, tranquilizers, or antidepressants were also excluded. The patients were subjected to preoperative assessment, and preoperative investigations [complete blood count, coagulation profile, liver and renal function tests, chest X-ray and electrocardiogram (if indicated)] were performed. All results were within normal values. The patients were admitted to the anaesthesia preparation room where monitors were applied (pulse oximetry, 5-lead electrocardiography, noninvasive arterial blood pressure), the heart rate (HR) and mean arterial blood pressure (MAP) were recorded before and after study drug administration, a wide bore intravenous cannula was inserted and crystalloid fluid infusion was started, and intravenous injection of 4 mg ondansetron was administered. However, sedative premedication was not given to the patients, and the patients were allocated randomly to three groups (*n* = 15 for each group) using sealed, opaque envelopes (indicating the group of each patient, carried out by an independent anaesthesiologist) to receive the study drugs (over 10 min) half an hour before the induction of general anaesthesia. The study solutions were prepared in identical syringes labelled “*study drug*”, and the anaesthesia residents who administered the study drug and who were in charge of general anaesthesia and collecting the data were blinded to the study protocol). **Group (C):** the control patients received 100 ml of normal saline IV. **Group (A1)**: the patients received 2 mg/kg intravenous (IV) aminophylline diluted in 100 ml of normal saline. **Group (A2):** the patients received 4 mg/kg intravenous (IV) aminophylline diluted in 100 ml of normal saline. In the operating room, monitors were applied (pulse oximetry, 5-lead ECG, end-tidal carbon dioxide, noninvasive arterial blood pressure, temperature probe and bispectral index (BIS) electrodes). Following preoxygenation, anaesthesia was induced by 2 μg/kg fentanyl, propofol infusion at 30 mg/kg/h was performed until a BIS value of 48 ± 2 was reached for 1 min (to ensure an adequate level of hypnosis) [12], and 1 mg/kg lignocaine and 0.5 mg/kg atracurium were administered to facilitate endotracheal intubation using a cuffed oral tube. Anaesthesia was maintained using 2% sevoflurane in an O_2_/air mixture, and mechanical ventilation was adjusted to maintain the end-tidal carbon dioxide at 36–40 mmHg. Normothermia was maintained by warming the IV fluids and using hot air convection. At the end of the surgery, the inhalation of sevoflurane was discontinued, and the neuromuscular block was reversed using 0.02 mg/kg atropine and 0.05 mg/kg neostigmine. Then, the patients were extubated, and after recovery, they were transferred to the PACU. For pain control, the following multimodal protocol was applied for all patients: at the end of surgery, IV paracetamol (perfalgan) at 1 g was administered, and infiltration of the surgical wound by bupivacaine hydrochloride (0.25%, 30 ml) was administered by the surgeon; no other opioids were administered to avoid affecting the ROC, extubation time or discharge time from the PACU once the patients were discharged to the surgical intensive care unit, where initiation of pain control was started according to the protocol planned by the SICU team.

The following data were recorded:

- Demographic data (age, sex, weight), ASA physical status (I or II) and duration of anaesthesia and surgery.
- Vital signs: Heart rate and mean arterial blood pressure were monitored continuously and recorded before and after study drug administration, after induction of general anaesthesia and then every 30 min for the duration of surgery.
- Adverse events after aminophylline administration (e.g., light-headedness, vomiting, chest discomfort, arrhythmia, hypotension or hypertension).
- The primary outcome: the ROC (in minutes), which is the time after discontinuation of anaesthesia until the response to a verbal command by eye opening, and time from propofol injection to the end point of hypnosis (defined as a sustained BIS value of 48 ± 2 for 1 min).
- The secondary outcomes: time for the BIS value to reach 80 after sevoflurane discontinuation [12], propofol dose (mg) until BIS reached 48 ± 2, time to tracheal extubation (in minutes) (which is the time from cessation of anaesthetic agent and recovery from neuromuscular blockage clinically and was monitored by a nerve stimulator), and time to discharge from the PACU (in minutes) (which is the time from arrival of the patient to the PACU till the modified Aldrete score reached ≥9 points).
- Intraoperative cardiovascular complications (e.g., sinus tachycardia: 20% increase in the heart rate from the baseline reading, hypotension or hypertension: 20% increase/decrease in the mean arterial pressure from the baseline reading, in case of tachycardia or hypertension the depth of anaesthesia was increased by increasing the concentration of the inhalational anaesthetics and administration of IV fentanyl at 50 μg, in case of hypotension; the concentration of the inhaled anaesthetic was reduced, and ephedrine was administered in 5 mg/kg increments, and causes of hypotension were excluded as intraoperative bleeding).
- The need for vasopressors or inotropes and fentanyl dose (μg).

### Statistical analysis

Sample size calculation was performed using the comparison of time to ROC, as reported in a previous publication [5]; the mean ± SD of time to ROC in normal saline group was 12.2 ± 4.73 min, and in the 6 mg/kg aminophylline group, it was 6.18 ± 3.96 min. No results were found in the published literature on lower doses of aminophylline. Therefore, we assumed that the effect of 6 mg/kg was similar to that of 4 mg/kg, and the effect of the 2 mg/kg dose was half that of the 4 mg/kg dose. The minimum sample size was 13 patients in each group to reject the null hypothesis with 80% power at the α = 0.05 level using a one-way analysis of variance test. The number of cases was increased to 15 in each group in case of a drop in cases. G*Power software version 3.1.2 for MS Windows, Franz Faul, Kiel University, was used.

Data are described in terms of the mean ± standard deviation or frequencies. Numerical data were tested for a normal distribution using the Shapiro Wilk test. Comparison of numerical variables was performed using one-way analysis of variance (ANOVA) with post hoc multiple 2-group comparisons to compare normally distributed data and Kruskal-Wallis tests with post hoc multiple 2-group comparisons to compare non-normally distributed data. Categorical data were compared by the chi-square (χ^2^) test. If the expected frequency was < 5, Fisher’s exact test was used. *p*-values < 0.05 were considered statistically significant, and *p*-values < 0.01 were considered statistically highly significant. Statistical calculations were performed using Statistical Package for the Social Science; IBM Corp, Armonk, NY, USA) release 22 for Microsoft Window.

## Results

**Fig. 1:**
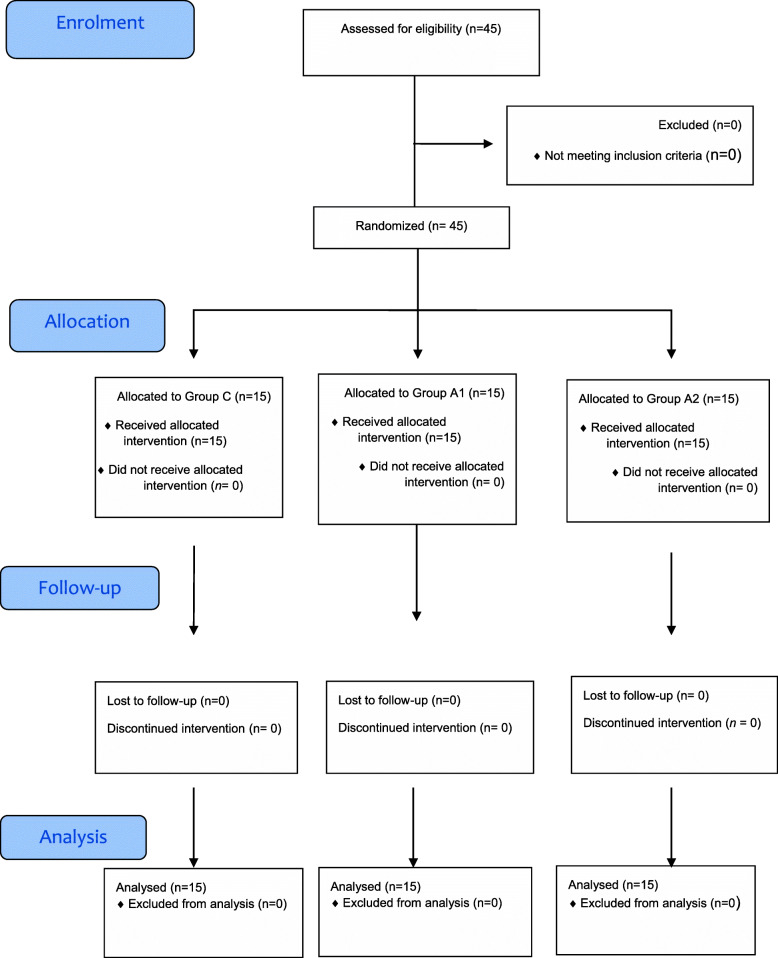
Participant flow diagram

The study included 45 patients who underwent pelvic-abdominal surgeries *[*e.g.*, abdominal exploration for intestinal obstruction, radical cystectomy, radical nephrectomy, splenectomy]*; all patients completed the study (Fig. 1).

There was no significant difference regarding the demographic data, ASA classification, duration of anaesthesia or surgery among the study groups (Table 1). The patients in the control group received lower doses of propofol and fentanyl than those in the aminophylline-treated groups, but this difference was not statistically significant. The time until BIS reached 48 ± 2 was highly significantly lower for the control group than for group A2 (70.67 ± 22.50 and 106.67 ± 34.77 *s for groups C and A2, respectively; p-value = 0.01)* (Table 1)**.**

**Table 1:** Demographic and operative data

	Group (C) ***(n = 15)***	Group (A1) ***(n = 15)***	Group (A2) ***(n = 15)***	*P* value
Age (yr)	45.07 ± 6.08	46.27 ± 8.70	42.73 ± 9.55	0.5
Weight (kg)	77.13 ± 10.04	77.07 ± 9.19	77.67 ± 21.28	0.99
Sex(M/F)	(9/6)	(6/9)	(10/5)	0.31
ASA (I/II)	(8/7)	(9/6)	(8/7)	0.91
Operation time (min)	176 ± 30.19	176 ± 23.84	176.33 ± 36.37	0.99
Anesthesia time (min)	195.33 ± 33.88	195 ± 20.96	194.4 ± 32.34	0.99
Propofol(mg)	176.67 ± 31.99	186 ± 21.64	190.67 ± 17.91	0.29
Fentanyl(ug)	185.33 ± 35.02	198 ± 31.21	212.67 ± 27.89	0.07
Time to BIS 48 ± 2 (sec)	70.67 ± 22.51	90.36 ± 34.77	106.67 ± 34.77	0.28^*^ 0.01^†^ 0.49^‡^

data are presented as mean ± standard deviation(±SD),or frequencies.*p between C and A1, † between Cand A2 and ‡between A1and A2

There was no statistically significant difference in heart rate or MAP among the studied groups *(p > 0.05)* (Tables 2 and 3, respectively)**.**

**Table 2:** Heart rate (beat/min)

	Group (C) ***(n = 15)***	Group (A1) ***(n = 15)***	Group (A2) ***(n = 15)***	*P* value
Before study drug administration	83.6 ± 17.37	83.2 ± 6.53	83.73 ± 6.54	1
After study drug administration	80.67 ± 11.75	80.2 ± 4.02	80.60 ± 4.54	1
After induction	81.40 ± 14.89	81.47 ± 6.40	80.60 ± 7.81	1
At30 min. of surgery	81.67 ± 16.67	82.33 ± 7.33	87.40 ± 16.99	0.84
At 60 min of surgery	80.2 ± 12.58	82 ± 6.84	80.73 ± 6.48	1
At 90 min of surgery	82.53 ± 9.31	82.13 ± 4.99	82.27 ± 6.75	1
At 120 minof surgery	83.60 ± 10.81	82.73 ± 5.72	83.40 ± 7.15	1
At 150 min of surgery	83.33 ± 13.55	83.80 ± 5.62	83.80 ± 4.63	1
At 180 min of surgery	83.87 ± 11.70	84.33 ± 6.41	84.00 ± 6.65	1

data are presented as mean ± standard deviation(± SD)

**Table 3:** Mean arterial blood pressure (mmHg)

	Group (C) ***(n = 15)***	Group (A1) ***(n = 15)***	Group (A2) ***(n = 15)***	*P* value
Before study drug administration	84.8 ± 17.35	84.47 ± 8.45	83.73 ± 6.64	0.96
After study drug administration	74.4 ± 12.25	75.13 ± 5.68	74.07 ± 7.68	0.94
After induction	66 ± 10.96	66.33 ± 6.02	66.33 ± 6.02	0.94
At 30 min. of surgery	80.93 ± 8.98	81.87 ± 6.49	82.40 ± 6.39	1
At 60 min of surgery	81.13 ± 7.13	82.20 ± 6.09	81.93 ± 9.06	1
At 90 min of surgery	81.80 ± 5.67	83.67 ± 6.14	83.40 ± 9.20	1
At 120 min of surgery	82.27 ± 4.72	82.93 ± 6.61	82.80 ± 9.56	1
At 150 min of surgery	82.73 ± 5.97	85.40 ± 6.91	84.00 ± 8.90	1
At 180 min of surgery	84.53 ± 6.49	85.20 ± 7.31	84.53 ± 7.41	1

data are presented as mean ± standard deviation(± SD)

The time until BIS reached 80 was significantly greater for the control group than the study group (5.6 ± 1.40, 3.5 ± 1.93 and 2.53 ± 1.72 min for groups C, A1 and A2, respectively, *p* < 0.01). The time to ROC was significantly longer for the control group than groups A1 and A2 (8.93 ± 0.92, 5.6 ± 2.47 and 4.53 ± 3.33 min for groups C, A1 and A2, respectively, *p*- value < 0.01). The extubation time (min) was longer for the control group than groups A1 and A2, and this difference was highly significant (12.4 ± 1.08, 7.87 ± 3.27 and 6.6 ± 2.47 for groups C, A1 and A2, respectively; *p* -value < 0.01), and the discharge time was not different among the study groups (*p* > 0.05) (Table 4).

**Table 4:** Recovery profile

	Group (C) ***(n = 15)***	Group (A1) ***(n = 15)***	Group (A2) ***(n = 15)***	*P* value
Time till BIS 80(min)	5.6 ± 1.40	3.5 ± 1.93	2.53 ± 1.72	< 0.01^*^ < 0.01^†^ 0.07^‡^
ROC (min)	8.93 ± 0.92	5.60 ± 2.47	4.53 ± 3.33	< 0.01^*^ < 0.01^†^ 0.14^‡^
Extubation time (min)	12.4 ± 1.08	7.87 ± 3.27	6.6 ± 2.47	< 0.01^*^ < 0.01^†^ 0.12^‡^
Discharge time (min)	31.67 ± 4.49	28.33 ± 6.47^*‡^	28.47 ± 9.72^†‡^	0.64^*^ 0.69^†^ 1.00^‡^

data are presented as mean ± standard deviation(± SD).*P between C and A1, † between C and A2 and ‡ between A1 and A2

No side effects related to aminophylline administration were reported, and none of the patients required intraoperative administration of vasodilators/vasopressors or inotropes.

## Discussion

The results of this study showed that premedication with aminophylline at 2 or 4 mg/kg (IV) reduced the time until BIS reached 80 after discontinuation of anaesthesia, time of ROC and extubation time without affecting the discharge time compared to those of the control group, and there were no significant differences between the groups treated with 2 or 4 mg aminophylline. Aminophylline is a nonselective adenosine receptor antagonist used for the treatment of asthma and chronic obstructive pulmonary disease [13]**,** and it has been used to antagonize the effects of anaesthetic and analgesic agents [14]. Its neuronal excitability is due to the inhibition of gamma-aminobutyric acid and central adenosine receptors [15]. Adenosine is involved in sleep and has a sedative and hypnotic effect [5, 16–18]. Its systemic administration enhances hypnosis induced by IV anaesthetics and decreases intraoperative anaesthetic requirements [19]. Perfusion of A1 receptor-selective antagonists such as caffeine or theophylline increased wakefulness, and in Turan et al. [5], the time to LOC was prolonged by aminophylline compared with saline in volunteers who were given 6 mg/kg IV aminophylline, followed by 1.5 mg/kg/h; the time to LOC was 7.7 ± 2.03 min for the volunteer group versus 5.1 ± 0.75 min for the saline group and the time to ROC was shorter (6.18 ± 3.96 min) for the volunteer group than the saline group (12.2 ± 4.73 min, *p* = 0.03). These findings are consistent with the results of this study. Turan et al. [5] also reported that the propofol dose at the time to LOC was greater in the aminophylline-treated group, and the researchers reported that the minimum bispectral index was greater with aminophylline than with saline (51 ± 15 vs 38 ± 9, *p* = 0.03). Turan et al. [3] concluded that recovery from sevoflurane anaesthesia and BIS scores improved in the early period when aminophylline was given at the emergence from anaesthesia. Ghaffaripour et al. [20] concluded that injection of aminophylline at emergence significantly increased BIS and reduced the recovery time in patients anaesthetized with intravenous anaesthesia. Hüpfl et al. [21] reported that the administration of aminophylline at 3 mg/k was associated with significant increases in BIS after discontinuation of sevoflurane anaesthesia for the control group compared to the study groups. The ability of aminophylline to decrease the anaesthetic effects of propofol can be explained by its antihypnotic effect or haemodynamic changes that affect the transfer of propofol to the brain [5]. Sakurai et al. suggested that the sedative/hypnotic effects of propofol can be explained by central adenosinergic mechanisms because ATP potentiates the sedative/hypnotic actions of propofol, while aminophylline reverses this effect [6]. Aghabiklooeiet [22] showed that intravenous aminophylline improved consciousness and respiration by antagonizing sedation induced by benzodiazepines. In this study, the extubation time was longer for the control group than the groups that received 2 or 4 mg/kg of aminophylline (12.4 ± 1.08, 7.87 ± 3.27 and 6.6 ± 2.47 for groups A, B and C, respectively; *p* -value =0.00), and the discharge time was not different among the study groups (*p* > 0.05). These results are consistent with those of Imani et al. [23], who concluded that the extubation times was decreased but not the discharge time for patients who received 1 or 5 mg/kg of aminophylline compared to the control patients after laparotomy. El Tahan [11] administered aminophylline after the end of surgery and reported that patients receiving 2, 3, 4 or 5 mg/kg aminophylline had shorter times to extubate and to home discharge than the controls. The differences between the results of the present study and other studies could be explained by the different doses of aminophylline and the time of its administration. Additionally, some of the previous studies were on volunteers, as in the study of Turan et al. [5]. Furthermore, Sakurai et al. [6] only included 2 cases where 5 mg/kg IV aminophylline was administered in the postoperative period to reverse propofol-induced sedation, and Turan et al. [3] examined cases with day surgeries.

### The limitation of this study

Due to the small sample size of this study, further studies are recommended in different surgical situations and with a larger number of patients to confirm the ability of aminophylline to enhance recovery and speed discharge from the PACU.

## Conclusion

Premedication with aminophylline at 2 or 4 mg/kg enhances the recovery profile after pelvic-abdominal surgeries under general anaesthesia compared to that of the control group, and cardiovascular complications were not reported.

## Data Availability

The datasets generated and/or analysed during the current study are not publicly available due to confidentiality of the Faculty of Medicine, Beni-Suef University files but are available from the corresponding author on reasonable request.

## Abbreviations

ANOVA: Analysis Of Variance
ASA: American Society of Anesthesiology
ATP: Adenosine Triphosphate
BIS: Bispectral Index
ECG: Electrocardiogram
HR: Heart Rate
IV: Intravenous
MAP: Mean Arterial Pressure
LOC: Loss Of Consciousness
PACU: Post-Anaesthesia Care Unit
ROC: Recovery Of Consciousness
SD: Standard Deviation
SICU: Surgical Intensive Care Unit
